# Scoping review of episodic future thinking and delay discounting observed in Alzheimer’s disease, depression, and PTSD

**DOI:** 10.3389/fpsyg.2026.1764387

**Published:** 2026-04-22

**Authors:** Jalyn R. Dubois, Esther Kim, Jennifer Pando-Bravo, Laura E. Martin, Richard Yi

**Affiliations:** 1 Cofrin Logan Center, University of Kansas, Lawrence, KS, United States; 2 The University of Kansas Medical Center, Kansas City, KS, United States

**Keywords:** Alzheimer’s disease, delay discounting (DD), depression, episodic future thinking, post-traumatic stress disorder

## Abstract

Delay discounting (DD), the decrease in the subjective value of a reward as its receipt is delayed, is associated with a variety of risky health behaviors. Episodic Future Thinking (EFT), the capacity to imagine events that might occur in the future, is understood as a reliable intervention in reducing DD. While populations known to exhibit heightened DD rates may benefit from EFT-based interventions, impaired capacity for EFT may reduce their effectiveness. This scoping review examines the available evidence regarding the co-occurrence of steep DD rates and impoverished EFT across Alzheimer’s disease (AD), post-traumatic stress disorder (PTSD), and depression to inform the transdiagnostic potential of EFT-based interventions. Searching and screening of articles were conducted on PsycINFO, PubMed, and Medline in February of 2025 following PRISMA guidelines. Across all disorders, clinical populations exhibited steeper DD rates, as well as impaired EFT characterized by marked reductions in specificity, detail, and novelty of the imagery. Cross-disorder comparisons highlighted reduced memory specificity impacting future simulation and physiological abnormalities in the hippocampus and vmPFC as potentially shared mechanisms in DD and EFT deficits. Despite a consistent pattern of steep DD and impoverished EFT occurring in each population and potentially influenced by similar mechanisms, the lack of studies examining DD and EFT in the same sample limits our conclusion and highlights where further research is warranted.

## 1 Introduction

Delay discounting (DD) refers to the decline in the subjective value of a reward as its receipt is delayed (Hudson et al., 2024; Odum et al., 2020; Bulley et al., 2016). High rates of DD, indicating a steep decrease in the subjective value of delayed outcomes, have been linked to shortsighted decision making representing a range of behaviors including but not limited to substance misuse, health-compromising behaviors, and poor financial planning (Petry, 2001; Snider et al., 2019; Odum et al., 2017). Many psychiatric populations consistently exhibit elevated DD rates compared to healthy controls (Amlung et al., 2019), and this preference for the immediate appears to represent a transdiagnostic mechanism underlying harmful decision-making tendencies (Hudson et al., 2024; Bickel et al., 2012; Brown and Stein, 2022).

Episodic future thinking (EFT) and related constructs (e.g., prospection, future event generation, simulation, future directed thinking, mental time travel) capture an individual’s capacity for projecting themselves into specific, imagined events that they might experience in the future (Hudson et al., 2024; Schacter et al., 2017; Hallford et al., 2018). EFT is relevant to multiple aspects of adaptive functioning, as it supports planning, emotion regulation, and goal-directed behavior (O’Donnell et al., 2017; Hallford et al., 2018). By projecting oneself into the future, an individual can effectively pre-experience an event and consider the consequences that could result from their decisions (O’Donnell et al., 2017).

EFT-based approaches have been validated as an effective and reliable method for attenuating DD (for a review see Ye et al., 2022). Engaging in EFT may widen individuals’ temporal window, facilitating future-oriented decisions (Snider et al., 2016) or a concrete, low-level construal of typically abstract, future outcomes (Yi et al., 2017). More importantly, reductions in DD following an EFT manipulation have been associated with healthier consumption among obese and diabetic populations, decreased self-administration of cigarettes in current smokers, and reduced alcohol demand in individuals with alcohol use disorder (Daniel et al., 2013; Stein et al., 2021; Stein et al., 2016; Snider et al., 2016; Brown and Stein, 2022). However, the efforts to apply EFT-based approaches as an intervention for clinical populations have been limited to date, and may be constrained by individual-level factors: For example, individuals with diminished capacity for vivid imagery appear to exhibit a blunted effect of EFT engagement on delay discounting (Peters and Büchel, 2010). So while populations known to exhibit elevated DD rates (Amlung et al., 2019; Godefroy et al., 2023) may benefit from an EFT-based intervention, impaired capacity for EFT and mental imagery poses a potential constraint on the efficacy of such interventions for such populations.

Given the potential of EFT-based interventions combined with current limitations in our understanding of their transdiagnostic utility, this scoping review sought to compare the existing behavioral and cognitive evidence of the co-occurrence of immediacy biases and EFT impairments in various clinical populations and identify gaps where further exploration is warranted. This review focuses on Alzheimer’s Disease (AD), depression, and post-traumatic stress disorder (PTSD), each representing a different clinical domain (neurocognitive, mood, and trauma, respectively) while displaying the equifinal outcomes of steep DD rate and impaired EFT despite their unique etiologies.

## 2 Methods

### 2.1 Search strategy

We follow a scoping review framework for this review, as it allows for the comprehensive summarization of a developing body of literature along with the identification of research gaps (Arksey and O'Malley, 2005; Harfield et al., 2018; Wagman et al., 2015). This review followed PRISMA guidelines to identify evidence on EFT impairments in each population. Three separate searches were conducted for each population to keep results digestible. PsycINFO, PubMed, and Medline were searched in February 2025, each utilizing the following search terms: Episodic future thinking OR Episodic thinking OR Episodic future OR Episodic generation OR Future generation OR Future simulation OR Future thinking OR Mental time travel OR Mental generation OR Prospection OR Prospective memory OR Mental Imagery OR Future directed thinking OR Future event OR Episodic specificity. Each search included specific terms to direct results towards specific populations. These terms are described in following sections. Using an iterative approach recommended for scoping reviews (Arksey and O'Malley, 2005), inclusion criteria for each disorder were determined post-hoc as the reviewers grew more familiar with the available evidence. General criteria for inclusion were as follows: (1) written in English, (2) published as a peer-reviewed scholarly article, (3) full text available, (4) methodology involving the generation of future, novel events, (5) study included a healthy control comparison, (6) if an intervention, baseline EFT characteristics were reported. Specific criteria will be described in following sections as well.

#### 2.1.1 Search 1—Alzheimer’s disease

Unique terms used in this search include dementia OR Alzheimer OR cognitive impairment. In addition to the general criteria, studies selected for inclusion in this review needed to examine a sample with a clinical diagnosis of Alzheimer’s Disease. The decision to focus on AD was made following the initial literature search, which indicates very limited research examining EFT in alternate forms of dementia and high variability across dementia subtypes.

#### 2.1.2 Search 2—depression

Unique terms in this search include depression OR depressed OR major depressive disorder. In addition to the general criteria, studies selected for inclusion in this review included participants with an isolated, clinically relevant level of depression. Projects that treated depression as a continuous variable or did not measure a depression-only group (e.g., studying groups with comorbid anxiety and depression) were excluded to isolate the barriers that depression poses to future thinking.

#### 2.1.3 Search 3—PTSD

Unique terms in this search include anxiety OR PTSD OR anxiety disorder. In addition to the general criteria, studies selected for review in this section included participants with a clinical diagnosis of post-traumatic stress disorder.

### 2.2 Data charting

Following full completion of all three searches, articles were reviewed and relevant data were entered into data charts using Microsoft Excel. The following characteristics were extracted for analysis: study design, sample characteristics, EFT measurement method used, EFT characteristics, and control comparison outcomes. EFT characteristics of interest included the following related to imagery: detail, specificity, vividness, novelty. Such features are relevant as they appear to influence the effectiveness of an EFT intervention, as those who report higher levels of imagery exhibit greater reductions of DD (Peters and Büchel, 2010).

In the context of EFT, detail refers to the contextual description, split into two categories (Hallford et al., 2018). Internal details refer to episodic details directly related to the main scene, whereas external details are considered superfluous (Hallford et al., 2018; Irish et al., 2012a). Degree of specificity is determined by the temporal and spatial exactness of the simulation (Hallford et al., 2018). The vividness of mental imagery reflects how clearly an individual pictures the future event in their mind (Liu et al., 2023). The novelty of imagined scenarios is necessary for guiding decisions, as they allow the individual to generate alternative scenarios and evaluate various approaches for upcoming events (El Haj et al., 2015b). We chose to focus on these core features as they are commonly considered by researchers as indicators of imagery quality and have been featured in past reviews (Hallford et al., 2018; Du et al., 2022). Additional characteristics such as the probability, plausibility, and subjective experience of future episodes were outside the scope of the current review.

Findings were synthesized narratively and represented visually via summary tables and Venn diagrams. We first discuss findings in delay discounting before moving on to EFT impairments in each disorder. Then, we examine overlap in the mechanisms driving deficits as potential evidence of co-occurrence.

## 3 Results

### 3.1 Alzheimer’s disease (AD)

#### 3.1.1 AD study characteristics

**Figure 1:**
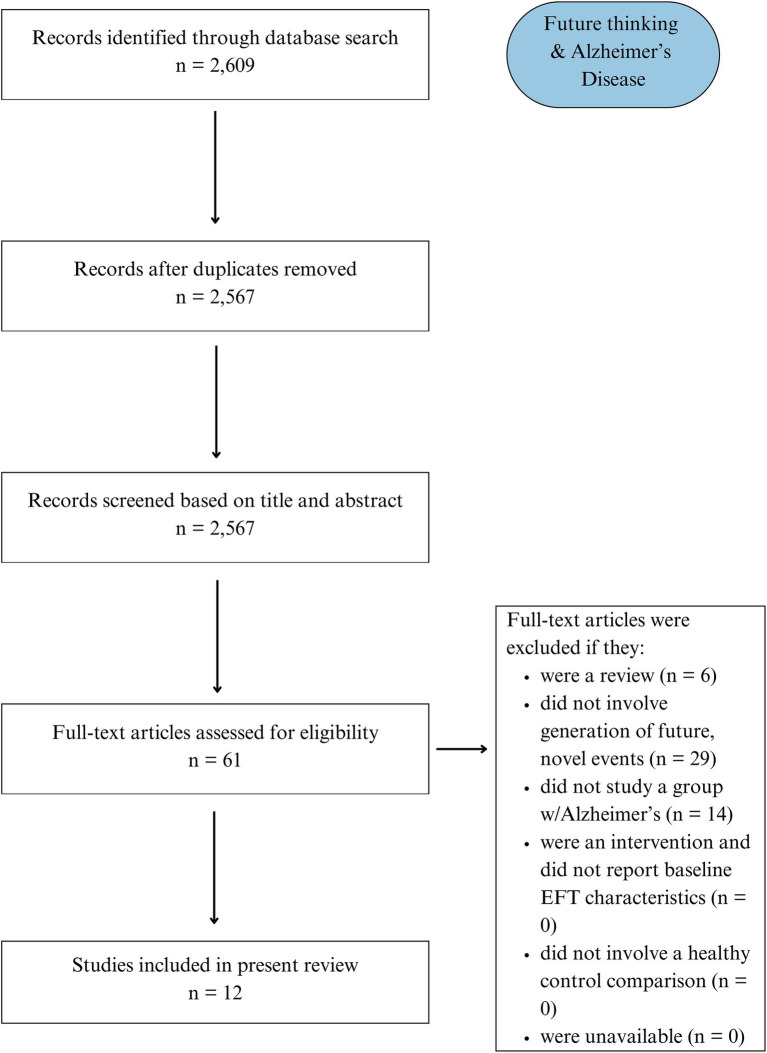
PRISMA flowchart detailing Search 1 (Alzheimer’s disease).

The initial search regarding episodic future thinking in Alzheimer’s Disease identified 2,609 records, which were reduced to 2,567 after removing duplicates. Records were then manually screened based on title and abstract. Of these, 61 full-text articles were further examined for eligibility. At the conclusion of this assessment, 12 studies met full inclusion criteria and were examined for our qualitative synthesis (Figure 1). Characteristics of included studies are presented in Table 1.

**Table 1:** Characteristics of studies included in Alzheimer’s and EFT synthesis.

Source	Sample characteristics	Diagnostic tool	EFT measure	Outcome
Irish et al. (2012a)	AD (11); HC (10)	Multiple interviews and neuroimaging evidence	Past-future task and autobiographical interview	AD generated fewer episodic details
Irish et al. (2012b)	AD (11); HC (14)	Multiple interviews and neuroimaging evidence	Past-future task and autobiographical interview	AD generated fewer episodic details
Irish et al. (2013)	AD (10); HC (10)	Multiple interviews and neuroimaging evidence	Past-future task and autobiographical interview	AD generated fewer episodic details
Irish et al. (2016)	AD (15); HC (20)	Multiple interviews and neuroimaging evidence	Memory and temporal experience questionnaire	AD were less correct in future imaginings
Addis et al. (2009)	AD (16); HC (16)	NINCDS-ADRDA diagnostic criteria	Past-future task and autobiographical interview	AD generated fewer episodic details
El Haj et al. (2015a)	AD (24); HC (26)	NINCDS-ADRDA diagnostic criteria	Autobiographical assessment	AD generated future scenarios that were more similar to past events than controls
Moustafa and El Haj (2018)	AD (28); HC (31)		Autobiographical assessment	AD group was less specific
El Haj et al. (2020b)	AD (40); HC (42)	Neurologist interview w/NIAAA criteria	Autobiographical assessment	AD group was less specific
El Haj et al. (2019)	AD (26); HC (30)	Neurologist Interview w/NIAAA criteria	Autobiographical assessment	AD group was less specific
El Haj and Antoine (2024)	AD (30); HC (32)	Neurologist interview w/NIAAA criteria	Autobiographical assessment	AD generated fewer episodic details, less specific
Glachet and El Haj (2020)	AD (24); HC (25)	Neurologist interview w/NIAAA criteria	Autobiographical assessment	AD generated fewer episodic details
El Haj et al. (2015)	AD (27); HC (30)	Neurologist Interview w/NIAAA criteria	Autobiographical assessment	AD generated fewer episodic details, scenarios were less novel

#### 3.1.2 Delay discounting and future-oriented decision-making in AD

Studies specifically examining DD among individuals with AD are very limited. Thus we expanded our review to identify studies examining *intertemporal choices* in AD. In general, the literature shows that individuals with AD consistently prefer immediate rewards compared to those without AD (Geng et al., 2020; El Haj et al., 2020a, 2020b; see Godefroy et al., 2023 for a review), though not all between-group differences reach statistical significance (Bertoux et al., 2015). Notably, a longitudinal study conducted by Thoma et al. (2017) examining DD in AD patients over a 2-year period found DD rates increased over time, suggesting that continued deterioration in the systems supporting cognitive functioning is associated with increased preference for immediate outcomes.

#### 3.1.3 EFT impairments in AD

Across numerous studies, individuals with AD consistently generated scenarios that were less detailed, specific, and novel compared to those without AD (Addis et al., 2009; Irish et al., 2012a, 2012b, 2013, 2016; Glachet and El Haj, 2020; El Haj and Antoine, 2024; El Haj et al., 2015b, 2019, 2020b; Moustafa and El Haj, 2018). Individuals with AD also provide less temporal and contextual information in their simulations (El Haj and Antoine, 2024), and exhibit a limited ability to imagine novel situations (El Haj et al., 2015b).

#### 3.1.4 Association between DD and EFT in AD

Research examining both DD and EFT concurrently in this population are lacking. Our search identified a single study directly examining both EFT capacity and DD in this population (El Haj et al., 2020b) In this study’s sample of 40 AD patients, less specific future events and higher DD rates (indicating a greater preference for immediate outcomes) were observed, compared to 42 healthy controls. Moreover, among participants with AD, a significant, negative correlation between EFT and DD was observed, indicating covariation of low future thinking ability and increased preference for immediate rewards. The authors speculate that decline in EFT interferes with the ability to project oneself into the future and fully consider the outcomes associated with decisions about the future.

#### 3.1.5 Neural and cognitive mechanisms of EFT in AD

Deficits in EFT can be connected to widespread atrophy in the neural network supporting mental time travel (Irish et al., 2012a; Irish et al., 2012b; Irish et al., 2016). EFT quality consistently correlates with atrophy in the posterior cingulate cortex (PCC) and the right posterior parahippocampal gyrus, with greater damage predicting greater deficits (Irish et al., 2012a; Irish et al., 2013). However, not all regions are so reliable. Studies show inconsistent, positive associations have been found with the frontal pole (Irish et al., 2012a) and the medial temporal regions (Irish et al., 2013), possibly reflecting heterogeneity in patient samples. Atrophy in these regions disrupts key cognitive processes supporting future projection. Damage to the hippocampus predicts reduced memory retrieval and cognitive flexibility (El Haj et al., 2015b). Memory retrieval is further attenuated by atrophy in the right frontal pole, PCC, precuneus, and temporal and medial lobes (Irish et al., 2012a, 2013). These findings suggest that EFT deficits in AD reflect the breakdown of a distributed network supporting essential functions of mental time travel. This implies that EFT is driven by multiple regions. Damage to any of these structures appears sufficient to disrupt EFT capability, even if the rest of the network remains relatively intact.

#### 3.1.6 Summary and research gaps (AD)

These observations regarding neural/cognitive processes observed in AD mirror those of Thoma et al. (2017), where rate of DD increased as over time and brain structures implicated in AD presumably degenerated. Taken together with the negative association between EFT quality and DD rate reported by El Haj et al. (2020b), it appears that impoverished mental time travel and a disproportionate preference for immediate rewards could be jointly produced by the breakdown of involved neural structures.

However, any conclusion of functional covariation is limited by the few studies examining both EFT and DD in this population, and an absence of studies that have explored a causal association. Moreover, we were unable to identify any longitudinal studies examining possible covariation of EFT quality and DD rate among individuals with AD or as a function of deterioration of brain structures. In order to firmly establish the association of EFT and DD among individuals with AD, more studies that replicate the effect observed in El Haj et al. (2020b) are necessary. Beyond that, the potential of an EFT-based intervention to reduce rate of DD in this population can be examined using methods similar to previous studies that seek to change DD using EFT approaches. Addressing these gaps in the literature would provide insights crucial to the potential implementation of EFT interventions targeting this population to address intertemporal decision making.

### 3.2 Depression

#### 3.2.1 Depression study characteristics

**Figure 2:**
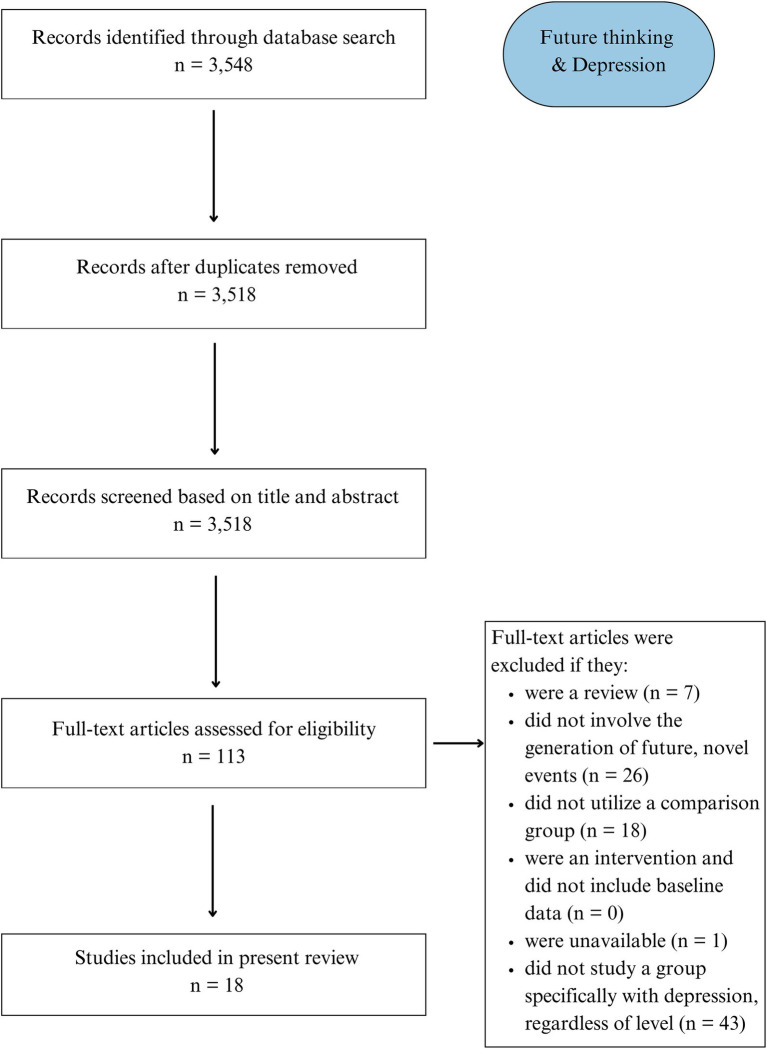
PRISMA flowchart detailing Search 2 (Depression).

Our preliminary search for studies examining episodic future thinking in depression identified 3,548 articles. After removing duplicates, 3,518 were screened for relevance based on title and abstract. Hundred and thirteen articles were assessed for eligibility. This resulted in 18 studies being included in the qualitative synthesis (Figure 2). Characteristics of included studies are presented in Table 2.

**Table 2:** Characteristics of studies included in depression and EFT synthesis.

Source	Sample characteristics	Diagnostic tool	EFT measure	Outcome
Hallford et al. (2020)	Current major depressive episode (117); Nondepressed controls (47)	Electronic-psychological assessment system and PHQ	Episodic future thinking-test	Depressed group was less specific, detailed, and vivid than controls.
Boland et al. (2018)	High dysphoria (24); moderate dysphoria (35); Nondepressed controls (38)	Center for epidemiologic studies depression scale revised	Future events prediction task and future simulation task	Positive events rated more vividly than negative, High dysphoric rated positive events as less vivid and negative events as more vivid than controls, similar pattern emerged for moderate dysphoria and controls, n.s.
King et al. (2011)	Major depressive disorder (22); Nondepressed controls (22)	DSM-IV	Modified Crovitz’s cue-word test	Depressed generated fewer episodic details
Yang et al. (2024)	Subthreshold depression (35); Nondepressed controls (35)	BDI-II and MINI	The prospection task	Depressed group was less specific.
Lavender and Watkins (2004)	Clinically depressed (30); Nondepressed controls (30)	SCID for DSM-IV	The future thinking task	Depressed group generated fewer positive events, equal in negative events.
Kosnes et al. (2013)	Subclinical depression (33); Nondepressed controls (38)	BDI-II	The future thinking task and future thinking-implicit relational assessment procedure	Depressed group generated fewer positive events, n.s.
Addis et al. (2016)	Current or past depressive symptoms (24); Nondepressed controls (24)	BDI-II	Autobiographical memory test–modified	Depressed group were less specific, generated fewer positive events
Bjärehed et al. (2010)	Depressed group (20); Nondepressed controls (20)	SCID for DSM-IV	The future thinking task	Depressed group generated fewer positive evets, equal in generating negative events
Anderson and Evans (2015)	Dysphoric group (31); Non-dysphoric controls (28)	Center for epidemiologic studies depression scale	Future event task	Dysphoric group was less vivid and detailed
Hach et al. (2014)	Depressed group (17); Nondepressed controls (16)	BDI-II	Autobiographical event tasks	Depressed group generated fewer future events and less detailed
MacLeod and Salaminiou (2001)	Depressed group (22); Nondepressed controls (22)	ICD-10	The future thinking task	Depressed group generated fewer positive events
Dickson and Bates (2006)	Dysphoric group (17); Non-dysphoric controls (17)	BDI	Future event task	Dysphoric group less specific
Belcher and Kangas (2014)	Depressed group (30); Nondepressed controls (30)	SCID for DSM-IV; BDI-II	Future imagining test	Depressed group less specific
MacLeod and Cropley (1995)	Dysphoric group (20); Non-dysphoric controls (24)	BDI	The future thinking task	Dysphoric group less specific to both positive and negative cues
Miles et al. (2004)	Depressed group (23); Nondepressed controls (42)	Children’s depression inventory short form	The future thinking task	Depressed group generated fewer positive events, n.s.
Han and Midorikawa (2024)	Depressed group (29); Nondepressed controls (37)	BDI-II	The future thinking task	Depressed groups generated fewer positive events, equal in negative events
Anderson et al. (2015)	Experiment 1–Dysphoric group (30); Non-dysphoric Control group (31) Experiment 2–Dysphoric group (27); Non-dysphoric group (26)	Center for Epidemiological Studies Depression Scale	Sentence completion for events in the future test	Depressed group less specific, generated fewer events, variable significance
Conaghan and Davidson (2002)	Depressed group (22); Nondepressed controls (22)	ICD-10	The personal future task	Depressed groups generated fewer positive events, equal in negative events

#### 3.2.2 Delay discounting and future-oriented decision-making in depression

A focused search for studies examining DD among individuals with depression indicates that this population consistently show increased preference for immediate rewards compared to healthy controls (for a meta-analysis, see Amlung et al., 2019). Imhoff et al. (2014) found a significant correlation between severity of depressive symptoms and rate of DD among adolescents: Higher scores on the Beck Depression Inventory was associated with higher rates of DD indicating relative preference for immediate rewards. Pulcu et al. (2014) discovered that patients currently experiencing depression discounted at a significantly higher rate than patients with remitted depression and healthy controls, who did not differ from each other. Hopelessness played a role as well, with a significant, positive correlation between severity of hopelessness and rate of DD. The authors connect their findings to Beck’s Cognitive Model of Depression – which characterizes depression as having a pessimistic view of the individual, the world, and the future - and conclude that immediate biases in this population are driven by an altered, negatively biased perception of time that increases the subjective cost of waiting for a delayed reward.

#### 3.2.3 EFT impairments in depression

Although depressive status varies across studies (e.g., clinically diagnosed major depressive disorder, experiencing a major depressive episode, various levels of dysphoria), individuals with depression consistently exhibit impaired EFT. Depressed participants generate future scenarios with less detail, specificity, and vividness compared to controls (Boland et al., 2018; King et al., 2011; Yang et al., 2024; Addis et al., 2016; Anderson and Evans, 2015; MacLeod and Cropley, 1995; Anderson et al., 2015) Further, they consistently generate fewer positive events compared to controls (Lavender and Watkins, 2004; Addis et al., 2016; Macleod and Salaminou, 2001; Han and Midorikawa, 2024; Conaghan and Davidson, 2002). In contrast, individuals with depression are as capable as those without depression in generating negative events (Bjärehed et al., 2010; Conaghan and Davidson, 2002; Han and Midorikawa, 2024). An adjacent body of literature examines how the construct of hopelessness contributes to EFT deficits in depression. A higher degree of hopelessness has been associated with reduced specificity (MacLeod and Cropley, 1995), and generation of smaller numbers of positive episodes (Conaghan and Davidson, 2002) and larger numbers of negative episodes (Han and Midorikawa, 2024). Taken together, the results of these studies suggest that EFT deficits in depression may relate to a cognitive bias towards negatively-valenced emotional content in future thinking, specifically supported by hopelessness.

#### 3.2.4 Association between DD and EFT in depression

Our initial search did not return any studies examining both intertemporal choices and EFT among depressed individuals, highlighting a major gap in the literature.

#### 3.2.5 Neural and cognitive mechanisms of EFT in depression

Neuroimaging indicates that depressed individuals show decreased activation of key structures. Depressed subjects show reduced activity and weakened connectivity between the right MTL (including the hippocampus), the mPFC, the PCC, and the left temporal pole when engaging in EFT (Hach et al., 2014). Cognitive functions supporting future event construction are weakened as well. For example, autobiographical memory is consistently overgeneralized, with past events recalled with reduced detail and specificity relative to controls (Belcher and Kangas, 2014; Anderson et al., 2015; King et al., 2011; Dickson and Bates, 2006). Moreover, deficits in strategic memory processing predicts lower specificity in future events (Addis et al., 2016). Notably, depressed participants were still capable of recruiting neural structures (Hach et al., 2014) and did not exhibit significant deficits in some non-EFT executive functioning compared to controls (Addis et al., 2016). This pattern suggests that even subtle declines in neural and cognitive functioning can degrade EFT quality.

#### 3.2.6 Summary and research gaps (depression)

Immediacy biases observed among this population appear to be driven by negatively biased expectations for the future (Pulcu et al., 2014). This characteristic pessimism provides a conceptual explanation for the difficulties individuals with depression show when generating positive/negative episodes, consistent with the increased hopelessness observed for compromised EFT and high rates of DD (Addis et al., 2016; Han and Midorikawa, 2024; Conaghan and Davidson, 2002; Pulcu et al., 2014).

However, firm conclusions regarding the relationship between EFT and DD in this population are speculative due to the absence of studies examining both processes concurrently. Additionally, the neural mechanisms engaged during DD among individuals with depression has not been explored. Filling these gaps would clarify the extent to which EFT and DD reflect shared mechanisms, informing the potential viability of EFT-based interventions aimed at modifying intertemporal decision-making in depression.

### 3.3 Post-traumatic stress disorder (PTSD)

#### 3.3.1 PTSD study characteristics

**Figure 3:**
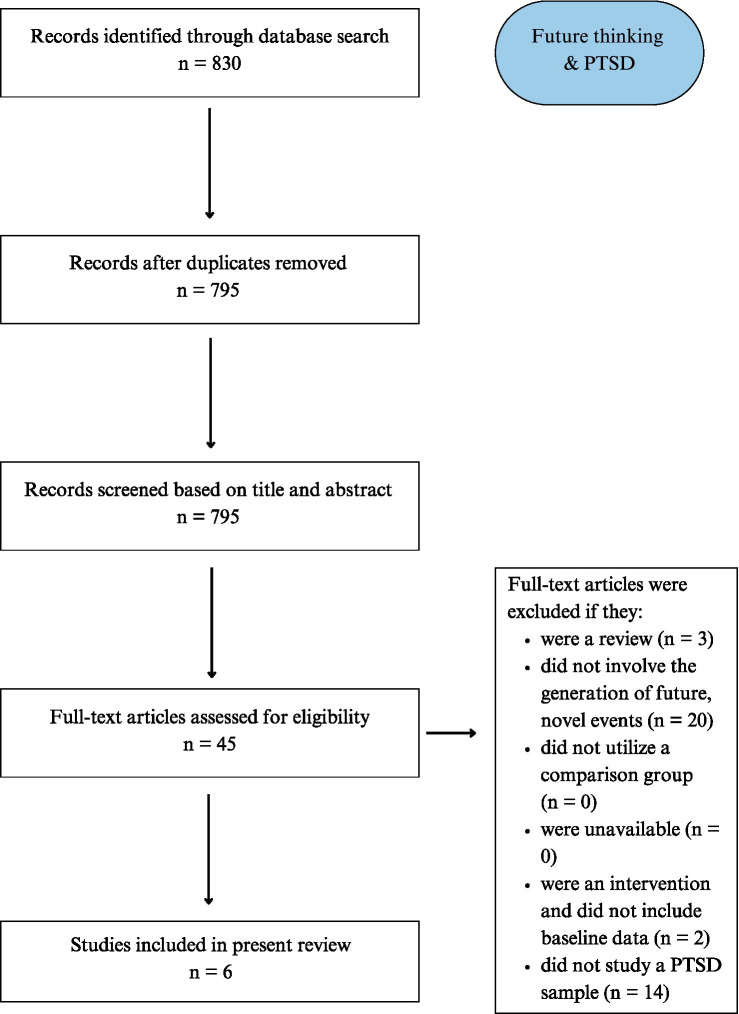
PRISMA flowchart detailing Search 3 (PTSD).

A comprehensive literature search investigating episodic future thinking in post-traumatic stress disorder initially identified 830 articles. After removing duplicates, 795 articles remained for further screening. Titles and abstracts were reviewed for relevance, resulting in 46 articles selected for full-text assessment. Following inclusion criteria, 6 studies were included in the final qualitative synthesis (Figure 3). Characteristics of the studies included are presented in Table 3.

**Table 3:** Characteristics of studies included in the PTSD and EFT synthesis.

Source	Sample characteristics	Diagnostic tool	EFT measure	Outcome
Brown et al. (2013a)	28 combat veterans (12 with PTSD, 16 without PTSD)	Clinician-administered PTSD scale (CAPS; Blake et al., 1995)	Autobiographical interview–episodic future thinking	Participants with PTSD generated less internal (episodic) details and more external (semantic) details
Brown et al. (2013b)	28 combat veterans (12 with PTSD, 16 without PTSD)	Clinician-administered PTSD scale (CAPS; Blake et al., 1995)	Autobiographical interview–episodic future thinking	Individuals with PTSD generated overgeneralized future events than individuals without PTSD
Kleim et al. (2014)	Trauma survivors (*N* = 50) – 30 PTSD, 20 non-PTSD	Clinician-administered PTSD scale (Blake et al., 1995)	Autobio- graphical memory test future (AMT-f)	Survivors with PTSD imagined fewer specific future event in response to positive, not negative cues.
Moradi et al. (2024)	Adolescents (*N* = 90) aged between 13 and 17 years (Mage = 15.53, SD = 1.13) who had been exposed to an earthquake in Iran and had (a) not developed PTSD (*n* = 30), (b) developed PTSD with low symptoms of depression (*n* = 30), and (c) developed PTSD with high symptoms of depression (*n* = 30)	Iranian Version of the structured clinical interview for DSM-5 disorders–clinician version (SCID-5-CV; Mohammadkhani et al., 2020)	Episodic Future Thinking Task	Individuals with PTSD had poorer performance on episodic future thinking than controls.
Verfaellie et al. (2023)	58 trauma-exposed U.S. military war-zone veterans: 25 individuals who met current criteria for PTSD (PTSDcurrent); 20 individuals with a lifetime history of PTSD who did not meet current diagnostic criteria (PTSDpast), and 13 individuals who had no lifetime history of PTSD (no PTSD)	Clinician Administered PTSD Scale for DSM-5	Adapted autobiographical interview (Lev- ine et al., 2002)	The current and past PTSD groups generated fewer internal details than the no-PTSD group across positive and negative cue words
Verfaellie et al. (2024a)	38 trauma exposed war-zone veterans with (*n* = 25) and without (*n* = 13) PTSD	PTSD symptom: Clinician administered PTSD scale for DSM-5, (CAPS-5; Weathers et al., 2018) Inclusion criteria: Structured clinical interview for DSM-5, research version	Future event fluency task	PTSD group generated fewer specific, but not generic, events than control

#### 3.3.2 4.3.2 Delay discounting and future-oriented decision-making in PTSD

Increased DD has been observed in large U. S. online adult samples with PTSD symptoms. Adults with more PTSD symptoms and a recent suicide attempt exhibit higher rates of DD compared to those with less PTSD symptoms (Bryan and Bryan, 2021). Moreover, trauma-exposed adults with PTSD symptoms exhibited steeper DD than trauma-exposed individuals without PTSD (Morris et al., 2020). A recent meta-analytic review indicates a small but significant positive association between post-traumatic stress and rate of DD among the 13 studies (Bird et al., 2024).

#### 3.3.3 EFT impairments in PTSD

Studies on individuals diagnosed with PTSD have demonstrated associations between PTSD and impaired future thinking, characterized by a reduced ability to generate vivid, specific simulations of future events (Brown et al., 2013a, 2013b; Kleim et al., 2013; Moradi et al., 2024; Verfaellie et al., 2023, 2024a). For example, Brown et al. (2013a) found that combat veterans with PTSD generated fewer internal (episodic) details in imagined future events than veterans without PTSD. Further, Verfaellie et al. (2023) demonstrated that veterans with PTSD produced fewer internal details of their imagined future events in response to positive and negative cues relative to controls. Brown et al. (2013b) found that, compared to veterans without PTSD, those with PTSD generated more overgeneralized future events, often incorporating combat-related content. Adolescents with PTSD also generated fewer specific future events than healthy controls (Moradi et al., 2024). Kleim et al. (2013) reported that when participants generated future events in response to positive or negative cue words, trauma survivors with PTSD imagined fewer specific future events following positive cues, but not to negative ones.

#### 3.3.4 Association between DD and EFT in PTSD

There is a dearth of research concerning both EFT and DD within the same study among individuals with PTSD. One exception examined the relationship between PTSD symptom severity and intertemporal choices (Verfaellie et al., 2024b). This study found that increased PTSD symptoms, particularly severity of avoidance, was associated with steeper DD. However, PTSD symptoms no longer predicted rate of DD following engagement in an EFT task. The authors further determined that difficulty in generating positive events mediated the relationship between PTSD symptom severity and DD. Specifically, greater PTSD symptom severity was associated with fewer specific positive events generated, which was linked to steeper DD.

#### 3.3.5 Neural and cognitive mechanisms of EFT in PTSD

Cognitive dysfunction associated with PTSD has been proposed to explain these impairments in future thinking. Disruptions in fear learning and memory processes lead to overgeneralized fear and failure to suppress fear responses towards non-threatening stimuli (Harnett et al., 2020). Individuals with PTSD also show deficits in episodic memory, working memory, and executive functions (Hayes et al., 2012). Overgeneralized autobiographical memory is also observed in PTSD individuals (Brown et al., 2013b). These cognitive features rely on the neural circuit involving the prefrontal cortex (PFC), hippocampus, and amygdala, all of which are affected in PTSD (Harnett et al., 2020). Considering that avoidance of trauma related stimuli is a core feature of PTSD (American Psychiatric Association, 2022; Bird et al., 2024; Verfaellie et al., 2024a; Verfaellie et al., 2024b), including trauma-related and negative simulations may further increase an individual’s avoidance tendency. However, the degree to which avoidance affects engagement with EFT remains as open questions.

Individuals with PTSD have been found to exhibit anatomical abnormalities in brain regions critical for memory, emotional regulation, and executive control. Structural abnormalities have been consistently observed in the hippocampus (comparatively smaller to non-PTSD subjects) which corresponds with impaired memory and contextual processing deficits (Fenster et al., 2018; Harnett et al., 2020; Karl et al., 2006). The anterior cingulate cortex (ACC) and vmPFC and dorsomedial prefrontal cortex (dmPFC) involved in emotional appraisal, reward valuation, and top-down regulatory control also show reduced volume (Duval et al., 2015; Harnett et al., 2020; Karl et al., 2006; Pulcu et al., 2014).

Studies have also demonstrated physiological deviations in brain function associated with PTSD. For example, hyperactivation is observed in the amygdala during exposure to trauma-related, emotional, and threatening stimuli, as well as during extinction tasks, indicating heightened emotional reactivity and fear processing (Fenster et al., 2018; Harnett et al., 2020; Hayes et al., 2012). The prefrontal cortex (PFC) shows different patterns of dysregulation depending on task demands. For example, Fenster et al. (2018) report increased prefrontal activity during sustained attention tasks, but reduced activation during reward stimuli and inhibition tasks. Harnett et al. (2020) note hypoactivation in the dorsolateral PFC (dlPFC) during anticipation of emotional stimuli and hyperactivity in dorsomedial PFC (dmPFC) during fear learning and extinction recall. These distinct patterns of PFC dysfunction may contribute to different PTSD symptom profiles. Likewise, the ACC responds differently depending on context: hyperactivation is observed in the ACC during threat processing (Duval et al., 2015), but hypoactivation during trauma-avoidance (Fenster et al., 2018) and symptom provocation (Kent and Rauch, 2003). This pattern highlights broader dysregulation in threat-processing and regulatory systems rather than one consistent abnormality.

Reduced activation in inhibitory control areas such as the medial prefrontal cortex (mPFC) during fear extinction tasks and emotional regulation tasks, and the vmPFC during reappraisal have also been comprehensively documented (Falconer et al., 2008; Fenster et al., 2018; Harnett et al., 2020; Hayes et al., 2012). This reflects diminished ability to regulate amygdala output and suppress fear responses and emotions. The dorsolateral prefrontal cortex (dlPFC) is similarly hypoactive during working memory and reappraisal (Brown and Morey, 2012; Falconer et al., 2008). Reduced hippocampal activation during memory tasks suggests memory and regulation deficits in individuals with PTSD (Fenster et al., 2018; Harnett et al., 2020).

#### 3.3.6 Summary and research gaps (PTSD)

Post-traumatic stress disorder is associated with steeper DD and impairments in EFT, including reduced specificity, diminished detail, and overgeneralized simulation, characterized by the tendency to generate categorical or repeated future events rather than unique moments within a distinct event. Neurocognitive evidence suggests that these impairments in brain regions supporting memory, emotional regulation, and executive control, including the prefrontal cortex, hippocampus, amygdala, and anterior cingulate cortex.

Despite these related findings, the association between EFT impairments and DD in PTSD remains unclear. Few studies have examined both processes together, and most existing work is cross-sectional. Future research assessing EFT and DD using concurrent and longitudinal designs would improve understanding of their relationship and inform the potential application of EFT-based approaches in this population.

### 3.4 Cross-disorder synthesis

**Figure 4:**
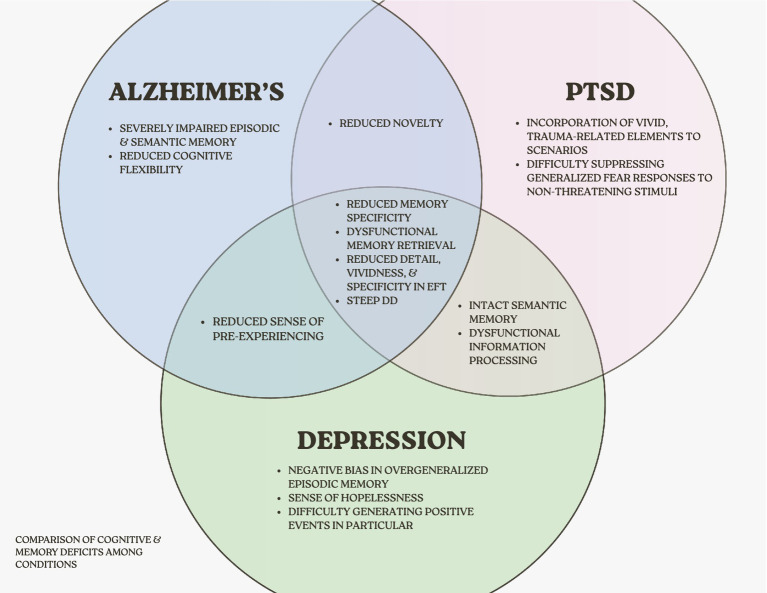
Comparison of cognitive & memory deficits among conditions.

Across the examined disorders, a consistent pattern emerges: Individuals with AD, depression, and PTSD exhibit both steeper rates of DD and EFT impairments, compared to healthy controls (El Haj et al., 2020b; Lebreton et al., 2013; Verfaellie et al., 2024b). The consistency with which this phenomenon occurs in each of these disorders despite their differing etiologies, neural dysfunction, and symptomatic profiles highlights the possibility of shared vulnerabilities that inform cross-disorder consideration (Figure 4). That said, the association *between* DD and EFT within these populations is constrained by the limited number of studies examining both concurrently.

#### 3.4.1 Cognitive mechanisms underlying impaired EFT across disorders

All three disorders show disruptions in the cognitive mechanisms that are integral for evaluating delayed rewards and simulating future events. Given linkages between past and future thinking, it is not surprising that low memory specificity predicts low level of detail in future thinking (El Haj et al., 2015b; El Haj et al., 2019; Brown et al., 2013a; Dickson and Bates, 2006; Belcher and Kangas, 2014). Reduced specificity in mental time travel undermines an individual’s capability to imagine the consequences of their decisions, a key process in DD (Godefroy et al., 2023). While EFT interventions typically do not instruct participants to simulate receiving the delayed reward, shifting attention toward a broader future outcome appears to guide present decision-making toward more adaptive choices that might support the imagined scenario. This may provide a potential, conceptual explanation as to why cuing episodic memories do not reliably reduce DD to the extent that EFT does despite their relationship (Lempert et al., 2017, 2024; Duff et al., 2025; Amlung et al., 2019; El Haj et al., 2015b). Related to specificity, memory retrieval is often dysfunctional in these populations as well.

AD patients display significant difficulties retrieving episodic memories (Irish et al., 2012a; Irish et al., 2013). In depression, strategic retrieval deficits were identified as a significant predictor of future event ambiguity (Addis et al., 2016). In PTSD, trauma-related memories interrupt future simulations, fueling avoidance and reduced novelty (Megías et al., 2007). As reduced autobiographical memory specificity is observed across AD, depression, and PTSD, the association between these disorders and DD/EFT may be due to diminished capacity to imagine the future consequences of their decisions or otherwise effectively pre-experience alternate outcomes of decisions (Godefroy et al., 2023). It is also possible that an underlying deficit in memory specificity, and the concurrent constraint in ability to imagine details of future events, contributes to a bias towards immediate/concrete outcomes (Trope and Liberman, 2010; Yi et al., 2017) in delay discounting.

#### 3.4.2 Neural mechanisms underlying impaired EFT across disorders

**Figure 5:**
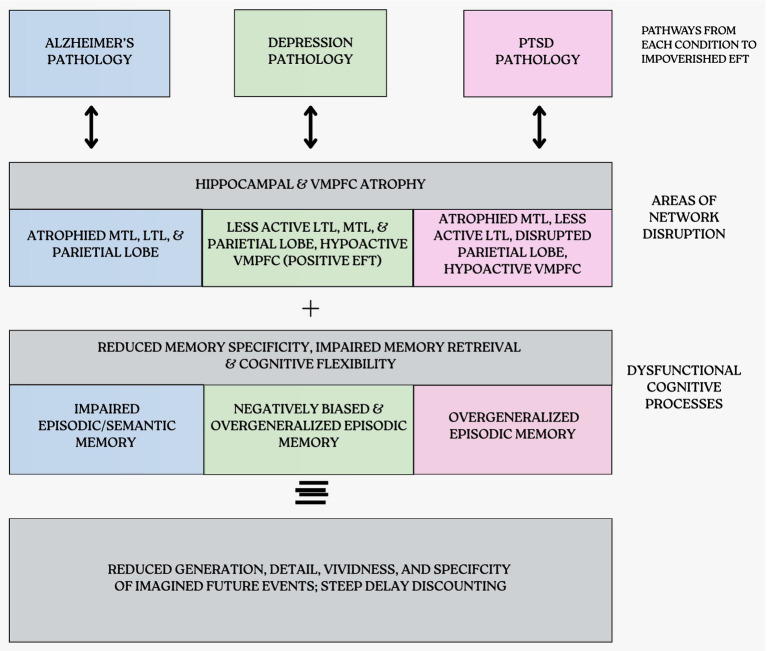
Pathways from each condition to impoverished eft.

Neural evidence shows that AD, depression, and PTSD all exhibit structural or functional irregularities in brain regions integral for DD and EFT, particularly the hippocampus and vmPFC. The hippocampus has been linked to retrieval, recombination of episodic memories, and construction of vivid future simulations (Schacter et al., 2017; Weiler et al., 2010; Race et al., 2011; Kurczek et al., 2015; Addis et al., 2011). The vmPFC facilitates projection of the self, retrieval of semantic memories, and signals the anticipated value of an event, informing choice selection (Schacter et al., 2017; Kurczek et al., 2015; Gaesser et al., 2013). Structural atrophy of hippocampus and vmPFC in AD (Irish et al., 2012a; Irish et al., 2013) constrain scene construction and valuation. In depression, reduced activation in these areas, especially while generating positive events, limit the ability to imagine a positive future (Hach et al., 2014). In PTSD, structural abnormalities in hippocampus correspond with impaired memory and contextual processing deficits (Squire and Zola-Morgan, 1991; Pitman et al., 2012; Smith, 2005), and hypoactivity in vmPFC impairs disrupt fear learning and memory processes. These shared structural and functional disruptions may underlie the observed EFT impairments across disorders, while their relationship to intertemporal choice remains as an important area for future research (see Figure 5).

Aside from these shared mechanisms, each disorder exhibits unique disruptions contributing to their concurrent EFT and DD impairments. In AD, progressive memory decline and reduced cognitive flexibility linked to structural atrophy in the hippocampus and vmPFC lead to less specific and less vivid EFT and reduced capacity to evaluate delayed rewards. In depression, hopelessness and a negative bias predicts a reduction in quality of imagined (positive) future events, and a reduction in willingness to wait for delayed rewards. In PTSD, overgeneralized memories and avoidance of trauma-related imagery are associated with the lack of future imagery, possibly contributing to reduced engagement in EFT.

## 4 Discussion

This scoping review was motivated by the emerging evidence that EFT may be an effective intervention for populations exhibiting high rates of DD and associated behavioral consequences. Thus we set out to evaluate evidence on steep delay discounting (DD) and impairments in episodic future thinking (EFT) across three clinical populations: Alzheimer’s disease (AD), depression, and post-traumatic stress disorder (PTSD). Across disorders, we identified a consistent pattern of greater preference for immediate rewards and impoverished future simulations (El Haj et al., 2020b; Lebreton et al., 2013; Verfaellie et al., 2024b). We highlight multiple areas of overlap that may explain these phenomena, as well as identify gaps in the research for future direction.

Deficits in autobiographical memory specificity and retrieval observed across these disorders appears to be a viable candidate for a shared mechanism underlying the co-occurrence of steep DD and impaired EFT. As memory functionality appears to be important in the construction and evaluation of the future, capacity to engage episodic memory may be instrumental in an individual’s ability to imagine and simulate future events. Moreover, across all three disorders, abnormalities in the hippocampus (supporting retrieval and recombination of episodic memories into coherent future simulations) and vmPFC (supporting self-projection, retrieval of semantic memories, and valuation of imagined outcomes) provide a neural explanation for these shared cognitive impairments.

Our review also identified several areas of the evidence base that are limited. Predominantly, there is a scarcity of simultaneous examinations of EFT and DD across disorders. Impoverished EFT and steep DD tendencies have been well established within AD, but examination of their association is limited, and AD patients’ responsiveness to an EFT intervention has not been explored. Within depressed populations, our search of the evidence base did not identify any study concurrently examining both EFT and DD. Amongst the populations we reviewed, the literature examining EFT and DD among those with PTSD is the most developed, though only one study examined both EFT and DD in PTSD (but did not specifically examine their association).

Consideration of methodological heterogeneity is also a factor in the evaluation of this body of research. For example, many studies are further limited by small samples. Moreover, variability in EFT measurement and DD paradigms limit cross-study comparability. And the predominance of cross-sectional studies and absence of longitudinal examinations leaves us unable to make conclusions regarding trajectories of EFT deficits and heightened DD nor the possibility of any causal mechanism. Finally, there is a lack of studies that examine how characteristic factors of pathological populations (such as medication use, symptom severity, or comorbidity) may affect our constructs of interest. Taken together, these limitations illustrate the need for systematic and longitudinal investigations before we can fully understand the relationship between mental time travel and intertemporal choices, as well as the clinical utility and transdiagnostic potential of EFT-based interventions to promote future-oriented decision-making.

Future research would benefit from implementing designs measuring both EFT and DD to further probe their relationship, to firmly establish the degree to these factors covary in these populations. Longitudinal studies should be conducted to assess trajectories and possible causal directionality. While many studies using EFT as an experimental manipulation or intervention indicate that engagement in EFT result in changes in DD, we are not aware of studies exploring the other direction of causation (changes in DD resulting in changes in EFT). Moreover, it is currently unknown whether populations reviewed here, with both high rates of DD and compromised EFT, would particularly benefit or not from an EFT-based intervention. That will likely depend on the etiology of the EFT deficit. If the deficit is due to limited regular use of EFT without any specific constraint, and that engagement in EFT by these populations will simply increase capacity (in the manner of strengthening a muscle), EFT-based interventions may work particularly well for the disorders reviewed here. However, if diminished engagement in EFT is due to limited capacity for EFT (e.g., organic causes, as might be with AD) without the possibility of compensatory mechanisms, EFT-based interventions will likely be ineffective in improving future prospection and subsequent changes in DD. A related question is whether EFT-based interventions should be disorder-specific or if a general EFT intervention is effective across disorders. That said, the developing body of literature exploring EFT as an intervention approach for disorders characterized by high rates of delay discounting appears to be a promising direction that informs not only the potential for EFT-based interventions to operate transdiagnostically, but also the exploration of a causal link between heightened DD and impoverished EFT.
